# Supplementary material for: Response of Antibiotic Resistance Genes and Related Microorganisms to Arsenic during Vermicomposting of Cow Dung
Source: Int J Environ Res Public Health. 2022 Nov 4;19(21):14475. doi: 10.3390/ijerph192114475 (PMC9658359; doi:10.3390/ijerph192114475)
Supplement: Supplementary file 1 [file ijerph-19-14475-s001.zip › ijerph-1966503-supplementary.pdf]

Table S1. Primers used in this study.

Primers		5'-3'Sequence
16S rDNA	Forword	CGGTGAATACGTTCYCGG
	Reverse	GGWTACCTTGTTACGACTT
*sul*1	Forword	CGCACCGGAAACATCGCTGCC
	Reverse	TGAAGTTCCGCCGCAAGGCTG
*sul*2	Forword	TCCGGTGGAGGCCGGTATCTGG
	Reverse	CGGGAATGCCATCTGCCTTGAG
*tet*L	Forword	GGTTTTGAAYGTYTCATTACCTGAT
	Reverse	GATAGCTTTCCATATASAGCTGTTCC
*tet*X	Forword	AGCCTTACCAATGGGTGTAAA
	Reverse	TTCTTACCTTGGACATCCCG
*tet*Q	Forword	AGAATCTGCTGTTTGCCAGTG
	Reverse	CGGAGTGTCAATGATATTGCA
*tet*O	Forword	ACGGARAGTTTATTGTATACC
	Reverse	TGGCGTATCTATAATGTTGAC
*tet*W	Forword	GAGAGCCTGCTATATGCCAGC
	Reverse	GGGCGTATCCACAATGTTAAC
bla_{ampC}	Forword	CCTCTTGCTCCACATTTGCT
	Reverse	ACAACGTTTGCTGTGTGACG
$bla_{\text{TEM-1}}$	Forword	CATTTTCGTGTCGCCCTTAT
	Reverse	GGGCGAAAACTCTCAAGGAT
$bla_{\text{OXA-1}}$	Forword	TATCTACAGCAGCGCCAGTG
	Reverse	CGCATCAAATGCCATAAGTG
bla_{NDM}	Forword	CAGCACACTTCCTATCTC
	Reverse	CCGCAACCATCCCCTCTT
*aad*A	Forword	GGTTATCGCCGAAGTATCAACTC
	Reverse	CACCGTAACCAGCAAATCAATATC
*fex*A	Forword	ATTCTCCCGCAAATAACG
	Reverse	TCGGCTCAGTAGCATCACG
cfr	Forword	TGTGCTACAGGCAACATTGGAT
	Reverse	CAAATACTTGACGGTTGGCTAGAG
*erm*C	Forword	GAAATCGGCTCAGGAAAAG
	Reverse	TAGCAAACCCGTATTCCACG
*erm*B	Forword	CCGATACCGTTTACGAAATG
	Reverse	TACTTTGGCGTGTTTCATTC
*qnr*B	Forword	GGMATHGAAATTCGCCACTG
	Reverse	TTYGCBGYYCGCCAGTCGAA
*qnr*S	Forword	GCAAGTTCATTGAACAGGGT
	Reverse	TCTAAACCGTCGAGTTCGGCG
*oqx*B	Forword	TCCTGATCTCCATTAACGCCCA
	Reverse	ACCGGAACCCATCTCGATGC
*str*A	Forword	TCAATCCCGACTTCTTACCG
	Reverse	CACCATGGCAAACAACCATA
*str*B	Forword	ATCGCTTTGCAGCTTTGTTT
	Reverse	ATGATGCAGATCGCCATGTA
*intl*1	Forword	GGCTTCGTGATGCCTGCTT
	Reverse	CATTCCTGGCCGTGGTTCT
*intl*2	Forword	TTATTGCTGGGATTAGGC
	Reverse	ACGGCTACCCTCTGTTATC
